# Work-Family Life Courses and Subjective Wellbeing in the MRC National Survey of Health and Development (the 1946 British birth cohort study)

**DOI:** 10.1007/s12062-015-9126-y

**Published:** 2015-07-09

**Authors:** Rebecca Lacey, Mai Stafford, Amanda Sacker, Anne McMunn

**Affiliations:** Department of Epidemiology & Public Health, University College London, 1-19 Torrington Place, London, WC1E 6BT UK; MRC Unit for Lifelong Health and Ageing, University College London, 33 Bedford Place, London, WC1B 5JU UK

**Keywords:** Wellbeing, Well-being, Work, Family, Gender, National Survey of Health & Development

## Abstract

Studies investigating the impact of combining paid work and family life on wellbeing have generally used information at one or a limited number of points in the life course, and have mainly focused on women. This study uses multi-channel sequence analysis to characterise work-family life courses across adulthood (ages 16–60) for more than 1500 men and women in the MRC National Study of Health and Development. Wellbeing at age 60–64 was captured by the Satisfaction With Life Scale (SWLS), Warwick-Edinburgh Mental Well Being Scale (WEMWBS) and the General Health Questionnaire (GHQ). A typology of 11 work-family groups was derived, across which there was greater variation for women. Adjusted for socioeconomic position, parental separation, adolescent internalising and externalising disorders, and health, men who had strong ties to paid work but no family had lower life satisfaction than those who combined work with parenthood and marriage (regression coefficient −2.89 (95 %CI: −5.04, −0.74); standard deviation for SWLS = 6.01). Women with weaker ties to paid work had lower life satisfaction, as did women who did not have children, compared to those who combined strong ties to paid work with marriage and parenthood. There were no significant associations between work-family life courses and WEMWBS or GHQ. This study shows that the way in which people combine work and family life may impact life satisfaction in early old age and highlights the need for policies that support combining work and family life.

## Introduction

The study of subjective wellbeing has been a growing area of research, particularly following the publication of the Stiglitz report (2009) recommending the use of markers of wellbeing in addition to GDP to motivate policy-making. Subjective wellbeing is an important predictor of health outcomes, such as lower mortality risk (Chida and Steptoe 2008; Koivumaa-Honkanen et al. 2000; Yoichi and Steptoe 2008), delayed onset of physical frailty (Ostir et al. 2004), slower cognitive (Gerstorf et al. 2007) and physical (Ostir et al. 2000) decline, as well as reduced physiological risk in terms of lower levels of pro-inflammatory cytokines, salivary cortisol, and fibrinogen (Ryff et al. 2004; Steptoe and Wardle 2005; Steptoe et al. 2005). Therefore, subjective wellbeing is of particular importance to older adults.

Subjective wellbeing is a complex, multi-faceted construct (Diener et al. 2003) which relates to both the evaluation and experiences of people’s lives (Stone et al. 2013). *Evaluative* wellbeing relates to life satisfaction (Diener 1984), whereas *experiential* wellbeing concerns emotional states, including both positive and negative affect (Stone et al. 2013). Optimal wellbeing is characterised by high levels of life satisfaction, high positive affect and low levels of negative affect. Organisations interested in population subjective wellbeing, such as the ONS and OECD, typically use multiple indicators capturing both evaluative (life satisfaction) and experiential (positive and negative affect) domains (OECD 2013; ONS 2014). This study therefore takes the same approach by including measures of affect and life satisfaction.

### Work-Family Life Courses and Wellbeing in Early Old Age

Strong ties to the labour market, as indicated by working full-time over long periods of the life course, have been associated with greater wellbeing and lower psychological distress (Clark et al. 2001; Di Tella et al. 2001; Dolan et al. 2008; Layard 2005; McKee-Ryan et al. 2005). Work provides opportunities for improved wellbeing through mechanisms such as increased social support networks, financial and material rewards (Waddell and Burton 2006). In Great Britain, like other Western countries, family life has historically limited women’s ability to participate fully in paid employment, with women more likely to take career breaks (Schober 2013; Woods et al. 2003). This is particularly true for the generations of women who are currently retirement age or older (McMunn et al. 2006). These women have spent less of their lives in paid work, partly due to the responsibilities of caring for young children in early adulthood and partly due to caring for parents, grandchildren or other family members in mid-life and early old age, both of which may be associated with poorer wellbeing (Dolan et al. 2008; Marks et al. 2002).

In addition to paid work, marital biographies characterised by long-term stable marriage were associated with better self-rated health and fewer depressive symptoms in the US Health and Retirement Study (Hughes and Waite 2009) and with higher life satisfaction in the German Socio-Economic Panel Study (Stutzer and Frey 2006). Evidence on the impact of parenthood on wellbeing is mixed (Kohler et al. 2005; Nomaguchi and Milkie 2003; Simon 2008), with some evidence that early entrance into parenthood is linked with poorer emotional wellbeing later in life (Liao 2003; Mirowsky and Ross 2002). Also there is a suggestion that life satisfaction in later life may be lower in those who did not have children (Connidis and McMullin 1993; Hansen et al. 2009).

In recognition of the interdependence of paid work, partnerships and parenting across adulthood, for women in particular but also increasingly for men, we examine these three life course domains in combination in relation to wellbeing in later life. Few studies have previously investigated the impact on wellbeing of work and family life courses in combination for both men and women. A limited number of longitudinal studies have specifically examined the health effects of work and family combinations among women, mainly suggesting that women who combine paid work with family responsibilities end up healthier than those who do not (Janzen and Muhajarine 2003; McKee-Ryan et al. 2005; Nordenmark 2004; Pavalko and Woodbury 2000), although not always (Johansson et al. 2007). Women who are now of retirement age or older and who were single and childless were more likely to be strongly attached to the labour market (Woods et al. 2003). Conversely women who were married and had a family were less likely than younger cohorts to be in paid work (Macran et al. 1996). Previous research on the cohort of women who are now of retirement age included in this study (the 1946 British birth cohort) showed that women who combined relatively strong labour market ties with stable marriage were subsequently healthier in their mid-50s than those who spent long periods of time out of the labour market looking after the home and family (McMunn et al. 2006). This study extends this work to later life using validated, multidimensional wellbeing indicators and also considers the impact of work-family life courses on the wellbeing of men in later life.

### Contribution of this Study

Previous research into the wellbeing consequences of work-family life courses has been limited by the lack of detailed longitudinal data which enables the derivation of detailed multi-domain work-family typologies. By employing a relatively novel statistical technique, multi-channel sequence analysis, this study derives work-family life courses which combine information on work, partnerships and parenthood. This approach takes the whole work-family life course as the unit of analysis and recognises these as interdependent life domains (Gauthier et al. 2010). Previous use of sequence analysis within a social science context has typically focused on one domain. The use of prospective longitudinal data means that prior wellbeing can be considered in the analysis. Much of the previous work on combining work and family roles has typically focused on women; however the consideration of men’s work-family life courses is becoming increasingly salient, particularly as men become more involved in childcare and family life. We therefore assess whether work-family life courses are important for the later life wellbeing of both men and women. Multiple measures of subjective wellbeing, both experiential and evaluative, are also used. The aim of this study is to investigate associations between work-family life courses and subjective wellbeing in later life of men and women of the MRC National Survey of Health and Development (NSHD, also known as the 1946 British birth cohort). It is hypothesised that work-family life courses characterised by strong ties to paid work, long-term stable partnerships and parenthood will have higher levels of subjective wellbeing.

## Methods

### Data

The NSHD was a stratified random sample of all births in Great Britain during a single week of 1946 (Wadsworth et al. 2006). Study members were all babies born to married women with husbands in agricultural or non-manual employment and one quarter of all comparable births to women with husbands in manual jobs (*n* = 5362). Sampling weights were used in this analysis to account for this stratification. Participants have been surveyed many times across their lives and information has been collected on economic, social, developmental and biological aspects at birth, on 15 occasions up to age 25 and at ages 26, 31, 36, 43, 53 and 60–64 years (Kuh et al. 2011). This paper uses information from across the life course from childhood through to age 60–64 years. The target sample of this latter survey (60–64 years) was 3163, the remainder of the initial 5362 full sample (2198) were not contacted for various reasons (567 (10.6 %) had emigrated, 594 (11.1 %) refused participation in previous waves, 718 (13.4 %) had died and 320 were untraceable since the previous survey at age 53) (Stafford et al. 2013). Of this 3163 target sample, 2661 (84.1 %) participated. Compared with UK census data, the sample somewhat over-represented those in paid work and married people in mid-adulthood (Wadsworth et al. 2003) and early older age (Stafford et al. 2013).

### Measures

#### Wellbeing

Three measures of wellbeing were collected when participants were aged 60–64. Diener’s Satisfaction With Life Scale (SWLS) comprises five positively-worded items^1^ tapping into life satisfaction and evaluative wellbeing (Diener et al. 1985). Each item has seven possible responses on a Likert scale (strongly disagree (1) to strongly agree (7)). Total scores were created by summing the five items (range: 5 to 35), with higher scores indicating greater life satisfaction. Secondly the 28-item General Health Questionnaire (GHQ) was used, capturing negative affect and symptoms of psychological disorder. Participants were able to respond via four possible response categories (‘not at all’ (0), ‘no more than usual’ (1), ‘rather more than usual’ (2) or ‘much more than usual’ (3)) to 28 statements of how they had been feeling over the past few weeks. Responses to all items were summed creating a total score (range: 0 to 84), with higher scores indicating more negative mental wellbeing. Finally the Warwick-Edinburgh Mental Wellbeing Scale (WEMWBS) was used as a measure of positive affect (Tennant et al. 2007). All 14 items^2^ are positively-worded and refer to feelings over the past fortnight. Responses ranged from ‘none of the time’ (1) to ‘all of the time’ (5). The responses from all items were totalled and high scores represent greater positive affect (range: 14 to 70). Internal consistencies of all three wellbeing measures were high (Cronbach’s alpha for SWLS: 0.90, GHQ: 0.90, WEMWBS: 0.91).

#### Work-Family Life Courses

Annual work, partnership and parenthood statuses were derived between ages 16 and 60 years. Work was categorised as full-time employment, part-time employment (≤30 h/week), homemaking or other not employed (sick, in education, unemployed, retired, not working for any other reason). Partnership status was coded as married, cohabiting, or not living with a partner. Parental status was categorised as no children in the household or youngest child >16 years, youngest child in household <5 years or youngest child in household 5–16 years. These three domains were combined into 44 combined work-family state variables (one for each year of interest between 16 and 60 years). Each variable had 36 possible combinations of work, partnership and parenthood (4 work states*3 partnership states*3 parenthood states).

Sequence analysis was used to derive a work-family life course typology. Sequence analysis compares each individual’s work-family sequence to all other individuals and additionally to a series of pre-defined ‘ideal types’. Eleven ‘ideal types’ based upon prior knowledge of this cohort were used in this study (Table 1). These ‘ideal types’ were chosen to reflect the most prevalent work-family combinations based on existing knowledge of the work and family lives of men and women in this cohort. For example, men and women largely partnered (married) early and made early transitions to parenthood. Men were mainly employed full-time from an early age and women were more likely to take work breaks to raise children. The work-family types chosen also varied in the timings in which key work and family transitions were made (e.g., entry into parenthood, retirement, work breaks).

**Table 1:** Work-family life courses types and their distribution in the NSHD

‘Ideal type’ labels	Description of ‘ideal type’ specification	% men	% women
‘Work, early family’	Continuous full-time employment; marriage and children from early 20s	33.9	6.9
‘Work, early family, retired’	Full-time employment, retired from age 55; early marriage and transition to parenthood	16.2	10.7
‘Work, later family’	Continuous full-time employment; cohabiting mid-20s, married from late 20s; children from early 30s	20.8	2.3
‘Work, later family, retired’	Full-time employment, retired from age 55; cohabiting mid-20s, married from late 20s; children from early 30s	10.3	1.6^a^
‘Work, marriage, non-parent’	Continuous full-time employment; married from early 20s; no children	7.8	7.0
‘Work, no family’	Continuous full-time employment; no partnership or children	7.9	5.1
‘Later family, work break’	Employed full-time until late 20s, homemaking from early-mid30s, employed part-time from late 30s, employed full-time from mid-40s; married from mid-20s; children from early 30s	0.5^a^	12.7
‘Early family, work break’	Employed full-time until early 20s, homemaking from early-late 20s, employed part-time early-mid-30s, employed full-time from late 30s; children from early 20s	0.6^a^	13.4
‘Part-time work, early family’	Employed full-time until early 20s, employed part-time from mid-20s; marriage and children from early 20s	1.2^a^	28.6
‘No paid work, early family’	Employed part-time until early 20s, homemaking from early 20s; marriage and children from early 20s	0^a^	7.0
‘Teen parent’	Homemaking until mid-20s, employed full-time from mid 20s; not living with a partner until early 30s, married from early 30s; children from late teens	0.9^a^	4.6

^a^Participants in groups with fewer than 2.0 % are not shown in subsequent analyses as there are too few participants for results to be reliable

**Fig. 1:**
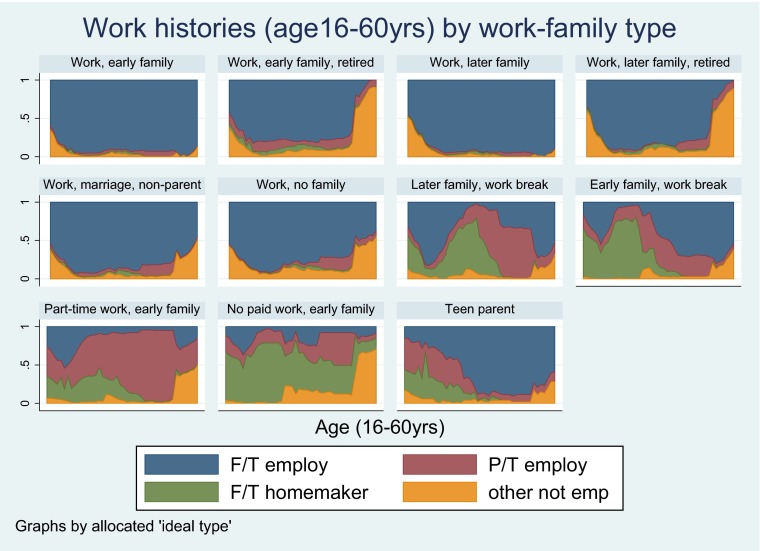
Work histories (16–60 years) by work-family type in the NSHD

**Fig. 2:**
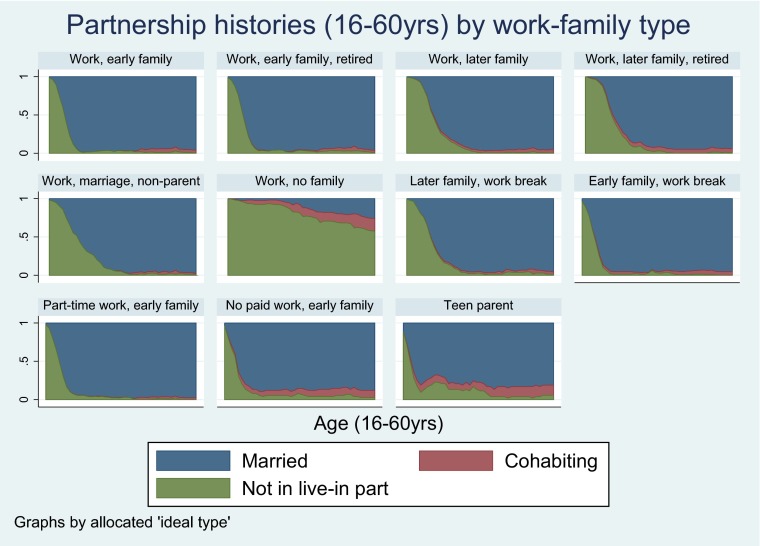
Partnership histories (16–60 years) by work-family type in the NSHD

**Fig. 3:**
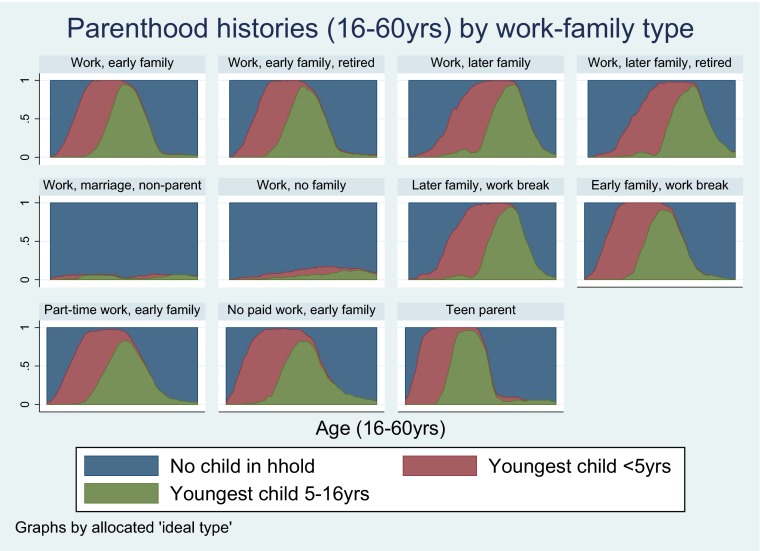
Parenthood histories (16–60 years) by work-family type in the NSHD

Additional work-family types were initially formulated but were dropped as few participants were found to occupy these groups. These included ‘Work, divorced parent’, ‘Work, divorced parent, early retired’, ‘Work, no family, early retirement’ and ‘Unstable work, no family’. Each participant was allocated to the ‘ideal type’ their observed work-family sequence was most similar to. The Dynamic Hamming algorithm was used, taking into account timing of transitions (Lesnard 2006). Dynamic Hamming requires sequences of equal length and with complete information, therefore missing information was multiply imputed using a method developed by Halpin (2012, 2013). Using this method 2000 participants had complete work-family information after imputation (imputed for 450 participants). Twenty imputed datasets were created. Between- and within-type distances were checked to assess the validity of the ‘ideal types’ chosen. More specifically, we assessed how close participants allocated to each work-family type were to the ‘ideal type’ that category was based on. Participants matched their ‘ideal types’ well (small variances in distances within each type). Also there were was no overlap between the mean distances and 95 % CIs of participants within each work-family type compared to other work-family types. Chronograms were used as an additional tool to check that participants had been appropriately allocated to their work-family types (see Figs. 1, 2 and 3).

#### Covariates

Indicators of early life socioeconomic position (SEP), prior wellbeing and parental separation were included in the analyses. More specifically childhood social class (Registrar General’s Social Class schema) was used to indicate childhood SEP. Father’s social class from age 4 was taken, where this was not known information was taken from age 7, and then from age 11 when age 7 information was additionally missing. Prior wellbeing was indicated by internalising and externalising disorders at ages 13 and 15. These were derived from pre-cursors of Rutter’s behavioural scales, reported by the cohort member’s teacher. Items relating to internalising problems included fearfulness, attention avoidance, anxiety, and shyness. Items capturing externalising problems included disobedience, lying, daydreaming, truancy, and restlessness. Overall measures of internalising and externalising disorders were derived by factor analysis of each and then categorising scores based on established percentile cut-points (Richards et al. 2009). For internalising scores: 0–50 % (absent), 51–87 % (mild) and ≥88 % (severe). For externalising scores: 0–75 % (absent), 75–93 % (mild) and ≥94 % (severe). Information on parental separation occurring in childhood was also included, as this is also known, on average, to have long-lasting influences on wellbeing (Amato and Keith 1991).

Educational attainment, derived as the highest qualification achieved by age 26, and social class of the head of household (Registrar General’s Social Class schema) at age 53, were used as indicators of adult SEP. To account for the correlation between physical and subjective wellbeing (Dolan et al. 2008) we considered the number of self-reported doctor-diagnosed physical health conditions reported at age 60–64 years which had been experienced since age 53 (the previous survey). Reported health conditions were stroke, diabetes, cancer and cardiovascular disease. Angina was additionally identified by the Rose/WHO angina questionnaire (Rose and Blackburn 1968). If information on angina or doctor-diagnosed health conditions was not available at age 60–64 it was taken from age 53.

### Statistical Analyses

Both the covariates and wellbeing outcomes were imputed in a second round of imputations using multiple imputation by chained equations. Von Hippel’s (2007) approach of multiple imputation and deletion was employed, whereby wellbeing measures were included in imputation models but only observed wellbeing data was used in subsequent analyses. Therefore of the 2000 participants with complete information on work-family life courses following the first round of imputation (see description above), the analyses are based upon 1518 participants with observed SWLS, 1681 with observed GHQ and 1509 with observed WEMWBS. Analysis of complete cases produced comparable results to those with imputed data (not shown). Descriptive results are presented for those with at least one observed wellbeing outcome (*n* = 1725).

Work-family types were cross-tabulated with wellbeing and covariates. Associations between work-family life courses and wellbeing were tested using linear regression in three separate models, controlling for all covariates. The ‘Work, early family’ type was used as the reference group. All analyses were stratified by gender as it is impossible to disentangle the effects of gender and work-family types, particularly in this cohort. Results for work-family types comprised of <2 % of men or women are not shown as estimates for these groups are likely to be unreliable (work-family types not shown are noted in Table 1). Two R-squared terms were estimated for each regression model, firstly for the overall variability in each wellbeing outcome explained by all analysis variables, and secondly the proportion of variability which could be attributed to the work-family types.

## Results

The distribution of men’s and women’s work-family life courses in this cohort is shown in Table 1. Almost all men (97 %) were in one of the work-family typologies characterised by continuous employment. One-third of men were allocated to the ‘Work, early family’ type, characterised by strong ties to full-time employment, long-term marriage and early transition to parenthood while a fifth of men were in the ‘Work, later family’ type, followed by 16 % in the ‘Work, early family’ and a further 10 % in the ‘Work, later family, retired’. Therefore most men in this cohort had work-family types comprised of multiple social roles which included strong ties to paid work – full-time employment, long-term partnerships and parenthood. As anticipated, women’s work-family life courses were much more diverse being more spread across the 11 types. The modal work-family type for women was the ‘Part-time work, early family’ work type (28.6 %), with the ‘Work break’ types next most common at 13 % of women in each. 11 % of women were in the ‘Work, early family, retired’ type, characterised by continuous full-time employment followed by early retirement, while the ‘No paid work, early family’ type was comprised entirely of women (7.0 % of total women).

Table 2 shows the descriptive statistics of all analysis variables by work-family type for men, as well as for the sample of all men regardless of work-family type. Table 3 shows the same analyses for women. Levels of SWLS and WEMWBS were the same for men and women (SWLS, means: men 27.3 (95 % CI: 26.9, 27.7), women 27.0 (95 % CI: 26.6, 27.4), WEMWBS: men 51.7 (95 % CI: 51.1, 52.2), women 51.9 (95 % CI: 51.3, 52.4)). Women reported higher GHQ scores than men (means men 15.1 (95 % CI: 14.6, 15.5), women 17.6 (95 % CI: 17.1, 18.2), *p* ≤ 0.001). Gender differences were seen in adolescent wellbeing, with women more likely to have had internalising disorders (12.3 % severe vs 8.4 % in men, *p* ≤ 0.001) and men to have had externalising disorders (6.5 % severe vs 4.9 % in women, *p* = 0.006). Women with weaker ties to paid work had more severe externalising and internalising problems in adolescence. Men with poorer adolescent wellbeing were more likely to retire early or not have family. Few participants (5.4 %) experienced parental separation or divorce during childhood. Men who experienced parental separation were more likely to be in the ‘Work, marriage, non-parent’ group and women who experienced this were more likely to be in the ‘Part-time work, early family’ and ‘Work, early family, retired’ types. Educational attainment by age 26 was higher for men compared to women, although around two-thirds of all participants had either no qualifications or school-leaving level qualifications. Men and women from more advantaged childhood and adult social classes, and who had higher educational qualifications were more likely to have work-family life courses characterised by later transitions to partnerships and parenthood, or be in the ‘Work, no family’ type (no children or partnerships but long-term full-time employment). Head of household social class at age 53 was higher for men compared to women (12.5 % social class I vs 8.1 % women, *p* = 0.009). Approximately three-fifths of the sample had no doctor-diagnosed stroke, cancer, cardiovascular disease, diabetes or angina and no one reported more than 3 of these conditions since age 53.

**Table 2:** Analysis variables by work-family life course types for men in the NSHD

	All men	Work,early family	Work, early family, retired	Work,later family	Work, later family, retired	Work,marriage, non-parent	Work, no family
Wellbeing (60–64 years)							
SWLS - mean (SD)^a^	27.3 (5.7)	28.1 (5.0)	27.4 (5.8)	27.1 (5.9)	27.2 (5.3)	26.8 (5.8)	24.9 (6.5)
GHQ – mean (SD)^a^	15.2 (6.8)	15.1 (6.6)	16.1 (7.5)	14.9 (6.5)	15.9 (7.8)	14.6 (6.0)	14.7 (6.9)
WEMWBS - mean (SD)^a^	51.9 (7.6)	52.3 (7.5)	52.2 (8.2)	52.4 (7.1)	50.1 (7.4)	51.3 (6.9)	51.0 (8.3)
Covariates							
Childhood social class	%	%	%	%	%	%	%
I	4.5	2.3	1.6	6.2	2.3	11.8	10.8
II	17.9	17.3	15.2	22.1	18.8	17.9	15.1
III non-manual	9.9	8.1	10.4	10.4	15.7	11.3	8.5
III manual	41.6	46.5	46.6	37.9	40.8	27.8	39.7
IV	18.1	18.5	20.5	13.6	12.8	22.8	17.1
V	8.2	7.3	5.7	9.9	9.6	8.4	8.8
Externalising problems (13, 15 years)							
Absent	74.7	68.1	79.8	75.7	82.4	78.5	77.9
Mild	18.8	24.6	12.8	19.5	9.7	17.2	18.3
Severe	6.5	7.3	7.5	4.8	8.0	4.4	3.8
Internalising problems (13, 15 years)							
Absent	58.6	64.0	63.9	49.2	56.8	60.2	46.9
Mild	33.0	30.6	26.3	43.6	31.7	29.1	38.0
Severe	8.4	5.5	9.8	7.2	11.5	10.7	15.1
Parental separation (0–16 years)							
No	95.7	96.2	98.0	95.4	94.5	91.6	95.9
Yes	4.3	3.8	2.0	4.6	5.5	8.4	4.1
Educational attainment (26 years)							
None	36.5	47.9	30.8	30.8	16.8	35.4	40.3
Vocational/O-level equivalent	20.8	19.7	26.0	19.9	28.0	14.5	19.1
A-level equivalent	29.5	23.4	30.4	32.9	39.1	33.1	23.9
Higher qual. or degree	13.2	9.0	12.9	16.4	16.1	17.0	16.8
Social class of head of household (53 years)							
I	12.5	10.0	4.0	17.1	24.9	15.5	7.9
II	38.6	35.3	41.0	41.3	49.7	28.6	34.4
IIINM	11.4	7.7	17.4	9.3	10.3	20.5	17.3
IIIM	26.7	31.2	25.2	25.2	13.5	24.5	29.0
IV	7.8	11.0	10.2	5.7	1.2	6.1	7.7
V	3.0	4.8	2.2	1.4	0.5	4.9	3.7
No. doctor-diagnosed health conditions (60–64 years)							
0	58.1	59.4	42.6	70.7	50.4	60.6	62.2
1	32.9	32.5	42.5	25.1	38.0	35.9	26.1
2	6.7	6.2	9.8	4.1	11.3	0	8.3
3	2.2	1.9	5.2	0.1	0.3	3.5	3.5

^a^Standard deviation NB there are fewer work-family types presented as groups containing <2 % of men (see Table 1) were excluded from subsequent analyses as there are too few for results to be reliable

**Table 3:** Analysis variables by work-family life course types for women in the NSHD

	All women	Work, early family	Work, early family, retired	Work, later family	Work, marriage, non-parent	Work, no family	Later family, work break	Early family, work break	Part-time work, early family	No paid work, early family	Unstable work, no family
Wellbeing (60–64 years)											
SWLS - mean (SD)^a^	27.3 (6.0)	28.9 (4.7)	28.3 (5.4)	28.3 (4.1)	26.6 (6.4)	25.4 (5.9)	26.8 (6.6)	27.7 (5.4)	27.1 (6.3)	26.0 (6.2)	27.5 (5.3)
GHQ – mean (SD)^a^	17.5 (8.8)	16.3 (8.2)	17.2 (9.0)	14.6 (6.7)	17.5 (7.4)	16.0 (7.5)	18.2 (8.7)	17.3 (9.3)	18.0 (9.7)	19.0 (7.6)	16.4 (7.1)
WEMWBS - mean (SD)^a^	52.2 (8.5)	53.3 (7.6)	52.7 (8.6)	53.5 (6.0)	51.9 (8.0)	51.4 (7.8)	52.5 (9.1)	53.1 (8.1)	51.1 (8.6)	51.6 (9.1)	52.5 (9.1)
Covariates											
Childhood social class	%	%	%	%	%	%	%	%	%	%	%
I	3.5	2.4	1.1	4.3	3.2	7.4	3.1	3.6	4.5	1.8	0.1
II	16.3	11.3	13.5	38.3	15.6	30.1	12.9	14.8	17.9	15.7	7.4
III non-manual	10.3	10.4	18.6	11.6	6.5	11.6	10.0	11.2	6.6	13.0	11.9
III manual	43.6	39.6	44.1	32.4	56.7	26.5	48.7	42.5	43.0	53.4	30.1
IV	19.6	33.5	19.3	13.4	9.4	16.5	23.0	18.3	18.8	11.1	38.8
V	6.7	2.9	3.6	0	8.7	8.0	2.4	9.7	9.3	4.9	11.7
Externalising problems (13, 15 years)											
Absent	80.1	79.7	76.8	87.0	86.9	77.6	82.3	81.9	80.3	67.4	79.3
Mild	15.0	13.4	15.3	11.4	10.1	17.1	17.1	15.8	12.1	26.9	18.3
Severe	4.9	6.8	7.9	1.6	3.0	5.3	0.6	2.2	7.6	5.7	2.4
Internalising problems (13, 15 years)											
Absent	46.4	49.6	56.3	65.2	59.9	35.7	35.1	46.5	46.8	36.9	46.2
Mild	41.3	42.0	36.5	30.5	30.8	54.6	46.3	47.0	39.3	44.4	37.9
Severe	12.3	8.5	7.3	4.4	9.3	9.7	18.6	6.5	13.9	18.7	15.9
Parental separation (0–16 years)											
No	93.7	96.7	90.4	100	95.8	95.2	95.9	94.4	90.0	95.0	99.7
Yes	6.4	3.3	9.7	0	4.2	4.8	4.1	5.7	10.0	5.0	0.3
Educational attainment (26 years)											
None	36.7	22.6	39.2	32.8	26.3	22.6	28.9	39.3	45.2	44.8	44.7
Vocational/O-level equivalent	39.3	47.2	30.2	31.3	50.3	33.6	41.5	43.9	35.0	38.1	50.0
A-level equivalent	20.1	29.2	25.5	30.4	20.4	31.3	25.3	14.0	15.7	13.6	5.2
Higher qual. or degree	3.9	1.0	5.1	5.5	3.0	12.6	4.3	2.8	4.1	3.5	0.2
Social class of head of household (53 years)											
I	8.1	8.9	6.0	13.7	2.9	3.1	14.4	10.0	8.2	5.5	1.1
II	39.4	30.7	38.1	61.8	45.9	42.3	47.6	36.1	33.4	47.5	39.5
IIINM	13.6	15.3	4.9	9.2	27.5	25.8	14.1	13.0	8.9	11.5	5.6
IIIM	23.6	23.0	20.4	6.4	9.5	21.9	14.3	25.1	33.4	19.5	33.5
IV	10.0	9.5	13.6	9.0	11.5	5.7	6.0	8.7	12.3	10.0	8.7
V	5.4	12.7	7.0	0	2.9	1.3	3.6	7.1	3.8	6.1	11.8
No. doctor-diagnosed health conditions (60–64 years)											
0	64.3	76.4	61.3	66.5	66.1	65.4	60.6	57.8	64.4	65.0	76.6
1	28.1	16.2	28.1	22.6	27.0	32.6	32.9	30.2	27.8	28.3	23.4
2	6.4	4.6	10.1	11.0	4.2	1.3	5.3	10.5	6.8	4.0	
3	1.3	2.8	0.5	0	2.8	0.8	1.2	1.5	1.0	2.8	

^**a**^Standard deviation NB there are fewer work-family types presented as groups containing <2 % of women (see Table 1) were excluded from subsequent analyses as there are too few for results to be reliable

### Associations Between Work-Family Life Courses and Subjective Wellbeing

Table 4 shows the multiply-adjusted association between work-family life courses and subjective wellbeing separately for men and women. Compared to those in the ‘Work, early family’ type, men who had strong ties to paid work but who weren’t partnered and didn’t have children (‘Work, no family’) had lower life satisfaction. When looking at the standardised estimates (not shown), this work-family type had the highest effect size compared to the effect sizes produced for other covariates in the model. Similarly, women who weren’t parents (‘Work, marriage, non-parent’ and ‘Work, no family’) also had lower life satisfaction compared to women in the ‘Work, early family’ type. The coefficient for the ‘Work, no family’ type for women was particularly large, equating to an average difference of >3 points (or >0.5 standard deviations) on the SWLS (Regression coefficient: −3.05, 95 % CI: −5.72, −0.39, p = 0.03). Also for women, work-family types characterised by weak ties to paid work (‘Later family, work break’ and ‘No paid work, early family’) had lower life satisfaction, on average, at age 60–64 compared to the reference group which had stronger ties to paid work (‘Work, early family’). Again for comparison, the standardised effect sizes were greater than for other covariates included in the model (not shown), with the exception of severe externalising disorders in adolescence. Around 2.8 % of the variability in SWLS for men and 9.8 % for women was explained by the covariates and work-family life courses. Most of this was attributable to the work-family life courses for men, but around 2.3 % was explained by this variable for women. No statistically significant association between work-family life course types and GHQ or WEMWBS scores were seen for women or men.

**Table 4:** Multiply-adjusted associations between work-family life course type and subjective wellbeing (SWLS, GHQ and WEMWBS)

SWLS	Men (*n* = 715)		Women (*n* = 803)	
	Regression coeff.^a,b^	95 % CI	Regression coeff.^a,b^	95 % CI
Work, early family	Ref		Ref	
Work, early family, retired	−0.29	−1.99, 1.42	−0.23	−2.21, 1.76
Work, later family	−0.60	−2.04, 0.84	−0.97	−3.45, 1.50
Work, later family, retired	−0.12	−1.71, 1.46	–	
Work, marriage, non-parent	−1.13	−3.13, 0.87	−2.70	−5.17, −0.22*
Work, no family	−2.89	−5.04, −0.74**	−3.05	−5.72, −0.39*
Later family, work break	–		−2.46	−4.64, −0.29*
Early family, work break	–		−1.15	−3.18, 0.88
Part-time work, early family	–		−1.54	−3.37, 0.28
No paid work, early family	–		−2.71	−5.19, −0.23*
Teen parent	–		−0.73	−3.27, 1.81
R-squared (%)	2.82		9.76	
R-squared attributable to work-family type	2.54		2.29	
GHQ	Men (*n* = 811)		Women (*n* = 870)	
	Regression coeff.^a,b^	95 % CI	Regression coeff.^a,b^	95 % CI
Work, early family	Ref		Ref	
Work, early family, retired	0.80	−1.15, 2.74	0.76	−2.58, 4.27
Work, later family	−0.08	−1.58, 1.43	−1.11	−4.52, 2.16
Work, later family, retired	0.96	−1.13, 3.05	–	
Work, marriage, non-parent	−0.41	−2.21, 1.40	1.31	−2.09, 4.41
Work, no family	−0.63	−2.76, 1.49	−1.33	−4.67, 2.83
Later family, work break	–		1.69	−1.44, 4.81
Early family, work break	–		0.26	−2.94, 4.14
Part-time work, early family	–		1.18	−1.60, 4.62
No paid work, early family	–		1.79	−1.54, 5.73
Teen parent	–		0.02	−3.72, 3.56
R-squared (%)	6.93		10.02	
R-squared attributable to work-family type	1.96		0.81	
WEMWBS	Men (*n* = 711)		Women (*n* = 798)	
	Regression coeff.^a,b^	95 % CI	Regression coeff.^a,b^	95 % CI
Work, early family	Ref		Ref	
Work, early family, retired	0.18	−2.23, 2.58	0.06	−3.63, 3.75
Work, later family	−0.16	−1.95, 1.63	−0.30	−4.91, 4.31
Work, later family, retired	−2.06	−4.43, 0.31	–	
Work, marriage, non-parent	−0.96	−3.15, 1.23	−1.93	−5.64, 1.78
Work, no family	−1.28	−4.00, 1.44	−0.75	−4.60, 3.09
Later family, work break	–		−0.90	−4.45, 2.64
Early family, work break	–		0.27	−3.15, 3.69
Part-time work, early family	–		−1.51	−4.62, 1.59
No paid work, early family	–		−1.58	−5.66, 2.49
Teen parent	–		0.43	−4.05, 4.91
R-squared (%)	7.98		6.27	
R-squared attributable to work-family type	1.68		0.91	

^a^Adjusted for childhood social class, parental separation, adolescent internalising and externalising disorders, educational attainment, head of household social class, number of doctor-diagnosed physical health conditions

^b^Participants in groups with fewer than 2.0 % are not shown in subsequent analyses as there are too few participants for results to be reliable

*p ≤ 0.05, **p ≤ 0.01, ***p ≤ 0.001

## Discussion

Using the 1946 British birth cohort we were able to investigate associations between detailed work-family life courses and subjective wellbeing in later life. In this study women who combined marriage and parenthood with little or no long long-term ties to paid work were found to have poorer wellbeing in later life, as captured by SWLS, even after accounting for prior wellbeing. Lower levels of health and wellbeing amongst homemaking women have been shown in many other studies (Janzen and Muhajarine 2003; Johansson et al. 2007; Kandel et al. 1985; Mirowsky and Ross 1989). This is also consistent with previous work using the same cohort which showed that long-term homemaking women had higher odds of poor self-rated health at age 53, than married women who had stronger ties to the labour market (McMunn et al. 2006). Our findings therefore suggest that differences in wellbeing extend to retirement age (60–64 years).

Strong ties to the labour market across the life course bring many benefits to wellbeing. Levels of wellbeing are known to be higher amongst those who are engaged in paid work than those who are not (Butterworth et al. 2011; Clark et al. 2001; Di Tella et al. 2001; Dolan et al. 2008). Work provides access to additional social support networks which are beneficial for wellbeing and provide added sources of support for stress from other life domains (Kandel et al. 1985; O’Driscoll et al. 2004). Work also provides access to additional financial resources as well as opportunities for learning and personal growth, both of which have been linked to higher wellbeing (Dolan et al. 2008; Waddell and Burton 2006). We have previously shown that strong ties to paid work for women are associated with better physical health profiles in mid-life in this cohort (McMunn et al. 2006). Full-time homemaking for long periods offers fewer opportunities to access these benefits and may offer one reason for lower levels of wellbeing in early old age relative to the ‘Work, early family’ type who combined full-time paid work with family. Also women in the 1946 cohort had their children in their early twenties (Kiernan and Diamond 1983); therefore, it is likely that most of their children would have left home by the time the women were middle-aged. The departure of children from the home has been shown to be a more stressful transition for mothers who do not work, compared to mothers who are employed outside of the home (Adelmann et al. 1989). Another explanation may be the uptake of new caring responsibilities. Informal caregiving, particularly to close relatives, has implications for wellbeing (Doress-Worters 1994; Hirst 2005; Marks et al. 2002), which may operate through loss of autonomy (Ryan and Deci 2001).

We additionally found that work-family life courses which were characterised by the absence of children (‘Work, marriage, non-parent’ and ‘Work, no family’) were associated with reduced life satisfaction in later life for both men and women. The findings from this study suggest that childlessness is associated with lower evaluative wellbeing in early old age. This supports previous work (Hansen et al. 2009; McQuillan et al. 2003), although the evidence base regarding the long-term influences of parenthood on wellbeing remains mixed (Dolan et al. 2008). The ‘Work, marriage, non-parent’ and ‘Work, no family’ types were only statistically different from the ‘Work, early family’ type on life satisfaction and not also on GHQ or WEMWBS. This finding supports previous work on the World Values Survey (Haller and Hadler 2006), whose authors suggest that being a parent puts demands on shorter-term emotions, such as those captured by the GHQ, but that children are an important part of cognitive evaluative wellbeing. Although not statistically dissimilar from men, the differences between ‘Work, marriage, non-parent’ women and ‘Work, early family’ women were particularly large (women: −3.05, 95 % CI: −5.72, −0.39; men: −2.89, 95 % CI: −5.04, −0.74). Childlessness represented a non-normative family type, particularly for women, in this cohort (Koropeckyj-Cox et al. 2007); the majority of NSHD participants had children by age 32 (84 % of women, 74 % of men) (Kiernan and Diamond 1983). However, we don’t know if participants were childless by choice or circumstance; childlessness by circumstance has been linked with lower wellbeing (Connidis and McMullin 1993). We may expect that our results relating those without children to lower wellbeing may be specific to this cohort, as childlessness becomes more normative. Differences in life satisfaction between the ‘Work, no family’ and ‘Work, early family’ types were particularly large for women, and were the only difference seen in life satisfaction for men. The ‘Work, no family’ type additionally did not involve long-term partnerships. Partnership histories characterised by long periods without a partner have been linked to reduced wellbeing in later life (Coombs 1991; Dykstra and Keizer 2009). Long-term stable partnerships bring benefits to wellbeing through emotional support and social integration, as well as financial and material benefits (Gerstel et al. 1985).

No statistical differences in WEMWBS or GHQ scores were seen between work-family types in this study. WEMWBS is known to be a mixed construct which captures positive affect as well as eudemonic evaluations (Tennant et al. 2007). This may be why there were null findings with this measure. One suggestion for future work would be to include separate measures of these domains. Work-family types were particularly associated with life satisfaction (an evaluative wellbeing measure), compared to the two experiential measures. This is consistent with previous research which showed that family and work-related life events had more of an effect on evaluative rather than affective wellbeing measures (Luhmann et al. 2012). Our findings are also consistent with work highlighting that positive mental wellbeing and mental ill health are distinct constructs and not merely opposite poles of a continuum (Ryff et al. 2006).

Men and women whose work-family types involved early retirement (‘Work, early family, retired’ and ‘Work, later family, retired’) did not have significantly lower subjective wellbeing than those in equivalent work-family types which were still working at age 60 (‘Work, early family’ and ‘Work, later family’). The evidence on wellbeing around the transition to retirement is mixed (Kim and Moen 2002; Westerlund et al. 2009). The lack of differences in our study may be because of heterogeneity in those who retire early. For instance, the more advantaged early retired may have greater wellbeing and the sick early retired are likely to have poorer wellbeing (Mein et al. 2000), resulting in a null difference overall. Also individuals who participated in full-time employment for most of their life course, particularly those in more advantaged social positions, will accumulate the benefits of work, as detailed above, which endure beyond retirement (Richardson and Kilty 1991). We were not able to account for reasons for retirement in this study and this would need to be investigated further in future work.

### Methodological Considerations

The importance of work, partnerships and parenting for wellbeing depends upon the quality and stress derived from these roles. It is likely that it is these factors, rather than work, partnership and parenting status, which affect later wellbeing (De Witte 2010; Diener 2000; Van der Doef and Maes 1999). We were unfortunately not able to take account of these factors in this study. We have considered the potential impact of work-family life courses on subjective wellbeing but acknowledge that the association is likely to be a bi-directional process. The ‘ideal types’ in this study were designed to be appropriate to both men and women, however UK state pension ages for men and women of this cohort, and similar ages, differ (60 years for women, 65 years for men). Early retirement was defined as 55 years in our ‘ideal types’. It is therefore possible that men allocated to our ‘Work, early family, retired’ and ‘Work, later family, retired’ types were more different from those still working than women allocated to these groups (10 years earlier retirement for men and 5 years earlier retirement for women). However wellbeing levels were found not to be statistically different between men and women allocated to these groups.

Despite these limitations this study uses a relatively novel statistical method - multi-channel sequence analysis - to give a holistic picture of work, partnerships and parenthood across adulthood and their importance for subjective wellbeing in early old age. We were also able to use more than one measure of wellbeing, capturing both evaluative and experiential aspects. Using longitudinal data we were able to account for the influence of early life factors, such as adolescent wellbeing, finding that work-family life courses were associated with later wellbeing even after accounting for prior wellbeing. We additionally accounted for missing data using a novel multiple imputation technique, therefore reducing the bias associated with attrition. Therefore the findings of this study are likely to be generalisable to adults, both men and women, of a similar age in Great Britain.

In summary, this study shows that long-term strong ties to a combination of full-time paid employment, parenthood and partnerships are associated with improved subjective wellbeing in early old age. Work-family life courses characterised by weaker ties to any one of these domains had lower levels of subjective wellbeing in their early sixties. These findings imply that policies which enable parents to maintain strong ties to paid work, such as affordable childcare and flexible working, are likely to improve wellbeing later into the life course.

## Footnotes

1 Diener SWLS items: 1. In most ways my life is close to ideal, 2. The conditions of my life are excellent, 3. I am satisfied with my life, 4. So far I have got the important things I want in life, 5. If I could live my life again, I would change almost nothing

2 WEMWBS items: 1. I’ve been feeling optimistic about the future, 2. I’ve been feeling useful, 3. I’ve been feeling relaxed, 4. I’ve been feeling interested in other people, 5. I’ve had energy to spare, 6. I’ve been dealing with problems well, 7. I’ve been thinking clearly, 8. I’ve been feeling good about myself, 9. I’ve been feeling close to other people, 10. I’ve been feeling confident, 11. I’ve been able to make up my own mind about things, 12. I’ve been feeling loved, 13. I’ve been interested in new things, 14. I’ve been feeling cheerful
